# Low-temperature derived temporal change in the vertical distribution of *Sesamia inferens* larvae in winter, with links to its latitudinal distribution

**DOI:** 10.1371/journal.pone.0236174

**Published:** 2020-07-28

**Authors:** Jianrong Huang, Guoping Li, Haixia Lei, Chunbin Fan, Caihong Tian, Qi Chen, Bo Huang, Huilong Li, Zhaocheng Lu, Hongqiang Feng

**Affiliations:** 1 Henan Key Laboratory of Crop Pest Control, MOA's Regional Key Lab of Crop IPM in Southern Part of Northern China; Institute of Plant Protection, Henan Academy of Agricultural Sciences, Zhengzhou, China; 2 Centre for Ecology and Conservation, University of Exeter, Penryn, Cornwall, United Kingdom; 3 Xinyang Academy of Agricultural Sciences, Xinyang, China; 4 Tianjing Beidagang Wetland Conservation Centre, Tianjing, China; 5 Luohe Academy of Agricultural Sciences, Luohe, China; Zhejiang University, CHINA

## Abstract

To escape or alleviate low temperatures in winter, insects have evolved many behavioral and physiological strategies. The purple stem borer, *Sesamia inferens* (Walker) is currently reported to be expanding their northern distributions and causing damage to summer maize in Xinxiang, China. However, their method of coping with the lower temperature in the new northern breeding area in winter is largely unknown. This paper investigates the overwinter site of *S*. *inferens*, and identifies the cold hardiness of larvae collected from a new breeding area in winter and explores a potential distribution based on low temperature threshold and on species distribution model MaxEnt. The results show that the overwintering location of the *S*. *inferens* population is more likely to be underground with increasing latitude and the population gradually moved down the corn stalk and drilled completely underground in later winter (February) in the north. The cold hardiness test shows the species is a moderate freeze-tolerant one, and Supercooling Points (SCP), Freezing Points (FP) and the incidence of mortality during the middle of winter (January, SCP: -7.653, FP: -6.596) were significantly lower than early winter (October) or late winter (March). Distribution in the new expansion area was predicted and the survival probability area was below N 35° for the Air Lower Lethal Temperature (ALLT_50_) and below N 40° for the Underground Lower Lethal Temperature (ULLT_50_)_._ The suitable habitat areas for *S*. *inferens* with MaxEnt were also below N 40°. This study suggests the overwinter strategies of *S*. *inferens* have led to the colonization of up to a five degree more northerly overwintering latitude.

## 1 Introduction

Temperature is the main abiotic factor that determines the growth and breeding of ectotherms. The distribution of most insects is directly related to extreme temperature [1]. The minimum temperature in winter which determines the survival rate of insect wintering populations is an important factor limiting the potential geographical distribution of insects [2, 3], particularly in ectotherms, setting northern range limits [3–5]. The low temperature in winter constrains the behavioral strategies of colonial insects [6], such as migration, drilling holes in refuges or making a thick cocoon, to avoid winterkilling. Insects also avoid chill injury by regulating physiological metabolism and accumulation of cold-tolerant substances, usually resulting in a lower Supercooling Point (SCP) of the overwintering population in mid-winter [7]. The warming of the global climate has also led to poleward distribution in some species' ranges, and may result in insects evolving new overwintering strategies in different breeding areas [8, 9].

The purple stem borer, *Seramia inferens* Walker, belongs to the order Lepidoptera, Noctuidae, and *Sesamia*. They are polyphagous insects and their hosts are mainly gramineous crops or weeds such as rice, wheat, maize [10]. With the changes in the farming system, crop distribution, and use of pesticides, *S*. *inferens* is gradually becoming a significant rice pest in many parts of China [11]. *S*. *inferens* is distributed in rice-producing countries, mainly in Asia. In China, the species is distributed south of N 34 degrees latitude [12]. The ranges are consistent with research by Ezcurra et al. [13]. Based on the cold resistant characteristics and field experiments of *S*. *inferens*, the distribution area was also agreed to be south of N 34 degrees latitude by Gu [14]. However, adult *S*. *inferens* were first found by light trap and also caused maize plant damage in Xinxiang City, Henan Province, China (E: 113.696°, N: 35.021°) in 2014 [15]. Further investigation illustrated that *S*. *inferens* can produce three generations in total and overwinter in local areas with mainly 3–4 larval instars [16]. They must have developed tolerance to cold to establish a population in the new northern breeding area in China in winter. Past research mainly focuses on overwintering in rice stubble in southern areas. It states that *S*. *inferens* overwinter as late instar larvae, and it is an exclusively freeze-tolerant insect [17, 18]. Significant damage to maize crops has also been reported in southern China [19]. However, studies on overwintering strategies of the pest in the maize planting area and cold tolerance of *S*. *inferens* in the new expansion area remain inadequate.

In this study, the behavioral strategies were confirmed by investigating the overwinter site and survival of *S*. *inferens* in early-middle-late winter in the field. The diapause larvae were also collected and the cold hardiness was measured, and a regional distribution based on low temperature threshold and species distribution model MaxEnt were used to generate a potential distribution of *S*. *inferens*. This research aims to identify the methods of *S*. *inferens* larvae to tolerate cold temperatures and to study the northern distribution boundary in the expansion zone.

## 2 Materials and methods

### 2.1 Latitudinal overwintering position of *S*. *inferens*

Field surveys were carried out and all *S*. *inferens* (dead or alive) larvae were examined in the late overwinter period on the 5^th^ of February 2016 in Nanjing (NJ) (32.65°N, 119.43°E) and the 5^th^ of February 2016 in Changsha (CS) (28.47°N, 113.35°E) in remaining rice stubble. The *S*. *inferens* population was also investigated on the 18^th^ of February 2016 in Xinxiang (XX) (35.02°N, 113.69°E) in remaining maize stalks. The number of *S*. *inferens* at vertical locations of two hosts was recorded. Two vertical positions of the remaining rice or maize stalk were devised: above ground (AG) and underground (10 cm deep). The data of Xianyou (XY) (25.36°N, 118.69°E) samples cited in Gu [20] were documented in a field survey on the 2^nd^ and 11^th^ of February in 2013.

### 2.2 Underground-forward behavior on corn stalks

The vertical distribution of *S*. *inferens* larvae in maize plants was destructively sampled to determine survival in field conditions during the winters of 2015–2016 on the 6^th^ of October, 2^nd^ of November, 10^th^ of December and 18^th^ of February in XX. We examined the large-scale remaining corn stalks. They usually were the same height, standing at 150 cm or a little over 150 cm, which meant the same positions of the stem stalk represented the same heights above the ground. The numbers of living and dead *S*. *inferens* larvae and the position of those insects in the maize stalk were recorded. Six vertical positions of the maize stalk, which remained completely in the field except the corn ear, from top to bottom are defined as positions 1–6. There are 4 positions (positions 1–4) above ground, and two positions underground (positions 5–6). Position 1 represents the high maize ear section (up to 120 cm). Position 2 represents two sections of the maize ear (70–120 cm). Position 3 represents two sections under the maize ear (30–70 cm). Position 4 represents three sections above the soil surface (0–30 cm). Position 5 represents the base of the maize stem and maize root under the soil surface about 10 cm deep. Position 6 represents the surrounding soil near the root, with a diameter of 10 cm.

### 2.3 Cold hardiness during winter

Wild *S*. *inferens* larvae were collected on remaining summer maize stalks in Xinxiang City (32°N, 119°E) in different winter periods on October 31^st^ and November 10^th^ in 2015, January 27^th^, February 18^th^ and March 30^th^ in 2016. Each larva replicate was placed in a 0.5 ml plastic tube with a 1–2 mm diameter ventilation hole in the lid and labeled. A weight test was carried out (Mettler Toledo ME204, Zurich, Switzerland). SCP and Freezing Points (FP) for each larval replicate were then measured by a small thermocouple thermometer [21] (Temp 20, Beijing Pengcheng Electronic Technology Center), which could automatically record the dropping temperature of an insect body per second. The SCP represents the first lowest temperature value of the body. After the SCP point, the body temperature jumps as the water within the insect cells starts to freeze, generating heat. The peak temperature represents the FP and the temperature begins to decrease after this. The thermometer was placed into the plastic tube, touching the larvae bodies, immobilized with cotton wool. Thermocouples were attached to a multichannel data logger (USB-TC, Measurement Computing, Norton, MA), and recorded and logged by using Tracer-DAQ software (Measurement Computing). The tubes were then placed in a -20°C freezer for 30 minutes. The body temperature of the larvae was gradually reduced, so that all larval replicates could have the SCP and FP recorded, as in Leather [22]. There were about 15 minutes from the test start to the reach point of the SCP and the cooling rate of the body was about 0.2–0.5°C/min. After testing, death was assessed by the lack of mandibular and body movement after 6 hours recovery at 24°C, and the incidence of mortality was identified by the chill-coma recovery numbers. All sample larvae were finally dried at 65°C for 48 h in a drying oven and then weighed again, to determine the water content of each larva.

### 2.4 Lethal low temperature experiment

Larvae were collected in Anyang (35.57°N, 114.85°E) in the same field on the 5^th^ of November 2015 and each larva was placed in a separate 0.5 ml plastic tube as mentioned above. Twenty centimeters of soil was placed in a container (L605×W410×H240 mm), along with moist cotton wool at the bottom of the container in order to guarantee the same humidity as the overwintering period. The tubes with larvae were then placed vertically into soil in the container. Two centimeters of soil was placed on the tubes to simulate the overwintering environment of *S*. *inferens* underground. The container was placed in an outdoor environment. Two microclimate sensors (TH11R, HHW) were installed in air (10 cm from the soil surface) with a shelf, and at 2 cm soil depth to record the temperature of both sites until March 30^th^ 2016. Low-temperature exposure experiments were carried out to measure the thresholds for the long-term survival of *S*. *inferens* at a constant temperature, separated into 4 times (Nov 9^th^, Dec11^th^, Jan 28^th^, Feb 19^th^) and seven gradient low temperatures (0°C, -5°C, -10°C, -15°C, -20°C, -25°C, and -30°C). All replicates of 140 in total were placed in a 0°C freezer for a 12 hour cold treatment, and then 20 replicates were taken out and placed at ambient temperature for 24 hours to determine whether they were alive or dead. The number of dead larvae was recorded. Those remaining replicates of 120 then continued to experience -5°C for 12 hours by reducing the freezer temperature, and then 20 replicates were taken out to check if alive, and so forth until all 7×20 larval replicates were tested. Ultimately, the incidence of mortality of different temperatures was identified. A low temperature threshold: Air Lower Lethal Temperature (ALLT_50_), which is defined as the temperature where 50% of individuals die in the low-temperature exposure experiments mentioned above, was calculated by a logistic linear regression [23]. In this study, the data of the sample dates were taken together to calculate the ALLT_50,_ and the ALLT_50_ value was -9.7°C.

### 2.5 Distribution based on low temperature threshold

Daily minimum temperatures in 47 different locations (Henan province, Hebei province, Tianjing city and Beijing city, formed a south-north belt in the north plain of China, in order to illustrate the northern overwinter site of *S*. *inferens*) from 2007 to 2014 were collected from national weather stations (https://data.cma.cn/). The winter temperatures from November to March were recorded in the air (10 cm from the soil surface) and underground (at 2 cm soil depth) by the two microclimate sensors mentioned above and it was found that there is an obvious significant linear relationship between lowest daily air and lowest underground temperature (*R*^2^ = 0.9161, *p*<0.001, *n* = 100). Based on this linear model, the minimum daily air temperatures in different locations were translated into the minimum daily soil temperature at 2 cm underground because the underground data were not available at national weather stations, and the landscape and soil composites of the 47 locations were very similar. Another low temperature threshold, Underground Lower Lethal Temperature (ULLT_50_) was also translated by linear equation and the value was -4.5°C, i.e. when the air temperature was -9.7°C the soil temperature postulated reached -4.5°C. Forty-seven location survival rates of air or underground in each year were speculated by predicting all larvae would die when the annual daily minimum temperature was lower than the ALLT_50_ or ULLT_50_, respectively, i.e. if the annual minimum temperature is lower than the ALLT_50_ or ULLT_50_, 0% of the population will survive. One hundred percent will survive if the annual minimum temperature is higher than the ALLT_50_ or ULLT_50._ The calculation of survival rates in different locations from 2007 to 2014, resulted in identification of the total proportion of insect survival over the 8 years. Based on the proportion of each location, a potential distribution based on annual minimum temperature threshold could be produced with ArcMap (version 10.2, ESRI, Redlands, CA, USA) [24].

### 2.6 The distribution based on MaxEnt

A species distribution model MaxEnt (version 3.3.3k) was used to generate a potential district-level distribution map of *S*. *inferens*. Nineteen bioclimatic layer data from the WorldClimte dataset were obtained [25] (http://www.worldclim.org/). Occurrences at 147 districts (locations) were obtained from published articles or websites, including the new northern breeding region in China [26], (S1 and S2 Tables). Sixty percent of total district (city) occurrences were used for model calibration (training data: 98 districts) and the remaining for model validation (test data: 49 districts). GoogleEarth was used to generate approximate coordinates. The area under the ROC (receiver operating characteristic) curve metric was used to evaluate the model performance [27, 28] (S1 Fig). Current and potential distribution maps were generated using ArcMap and three arbitrary categories were defined as low (<0.15), medium (0.15–0.53) and high (>0.53) based on predicted habitat suitability.

### 2.7 Statistics

Data were tested for normal distribution using the Shapiro-Wilk test and normally distributed data was compared by an independent samples t-test and Pearson’s correction test. The differences in the incidences of mortality exposed in different sub-zero temperatures between different sampling periods in winter were compared with two factors across anaylsis of variance. The differences between factors were evaluated using Tukey-Kramer groupings comparison in the least squares means and Non-normally distributed data were compared by nonparametric tests of group differences. The linear or nonlinear models were identified with p<0.05 for the significance, and all data were analyzed with the software program R 3.5.3 (R Core Team 2017).

## 3 Results

### 3.1 Geographical variation in overwinter position

**Fig 1:**
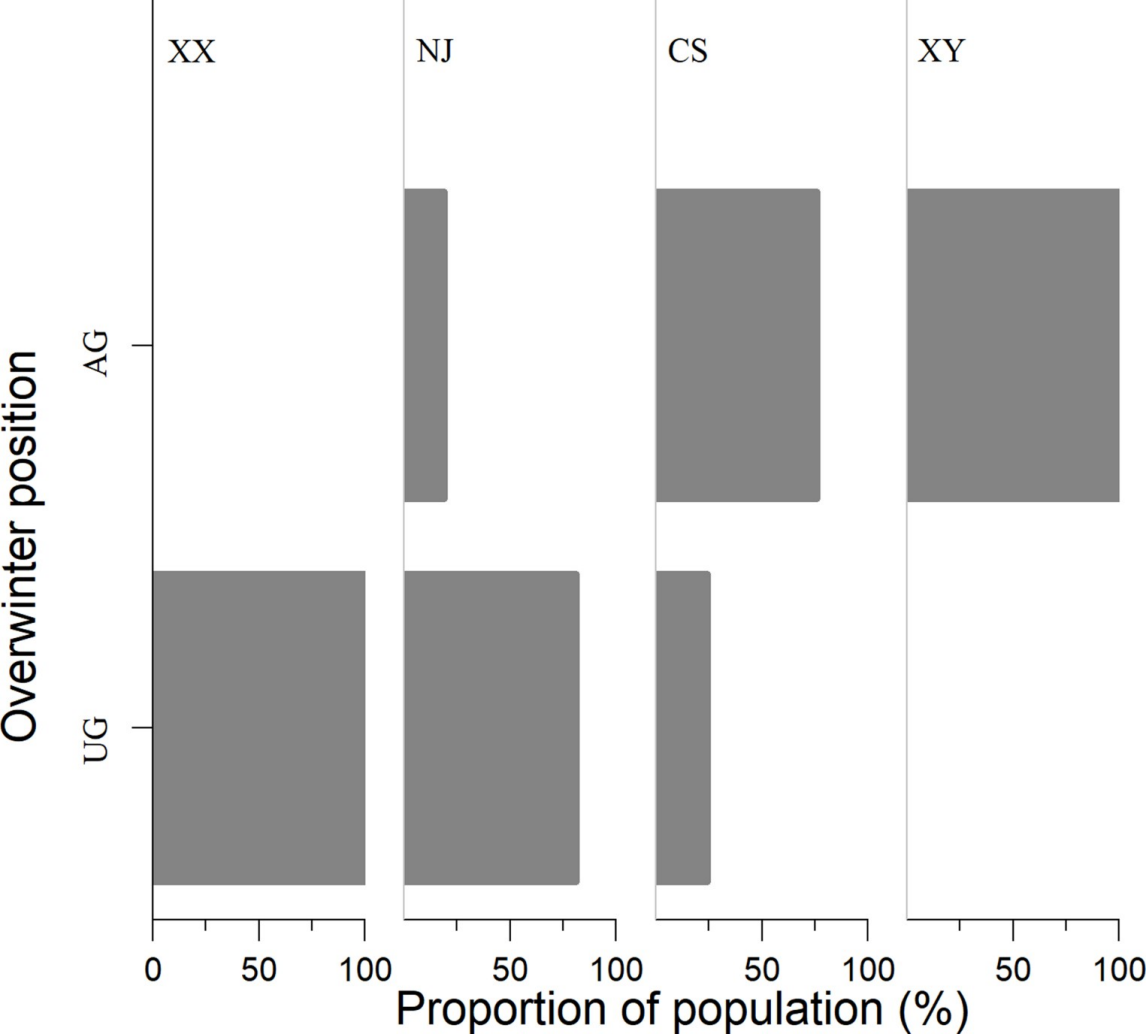
Geographical variation of living *S*. *inferens* population in overwintering position. UG: Underground; AG: above ground, XX: Xinxiang(*n* = 24), NJ: Nanjing(*n* = 74), CS: Changsha(*n* = 33); XY: Xianyou(*n* = 13).

The living *S*. *inferens* population was closer to the ground with increasing latitude in late winter and 100% and 75% of overwinter individuals were underground in northern locations in XX and NJ, respectively. But in southern regions, CS and XY, they were mainly above ground (Fig 1).

### 3.2 Overwintering behavioral in maize stalk

**Fig 2:**
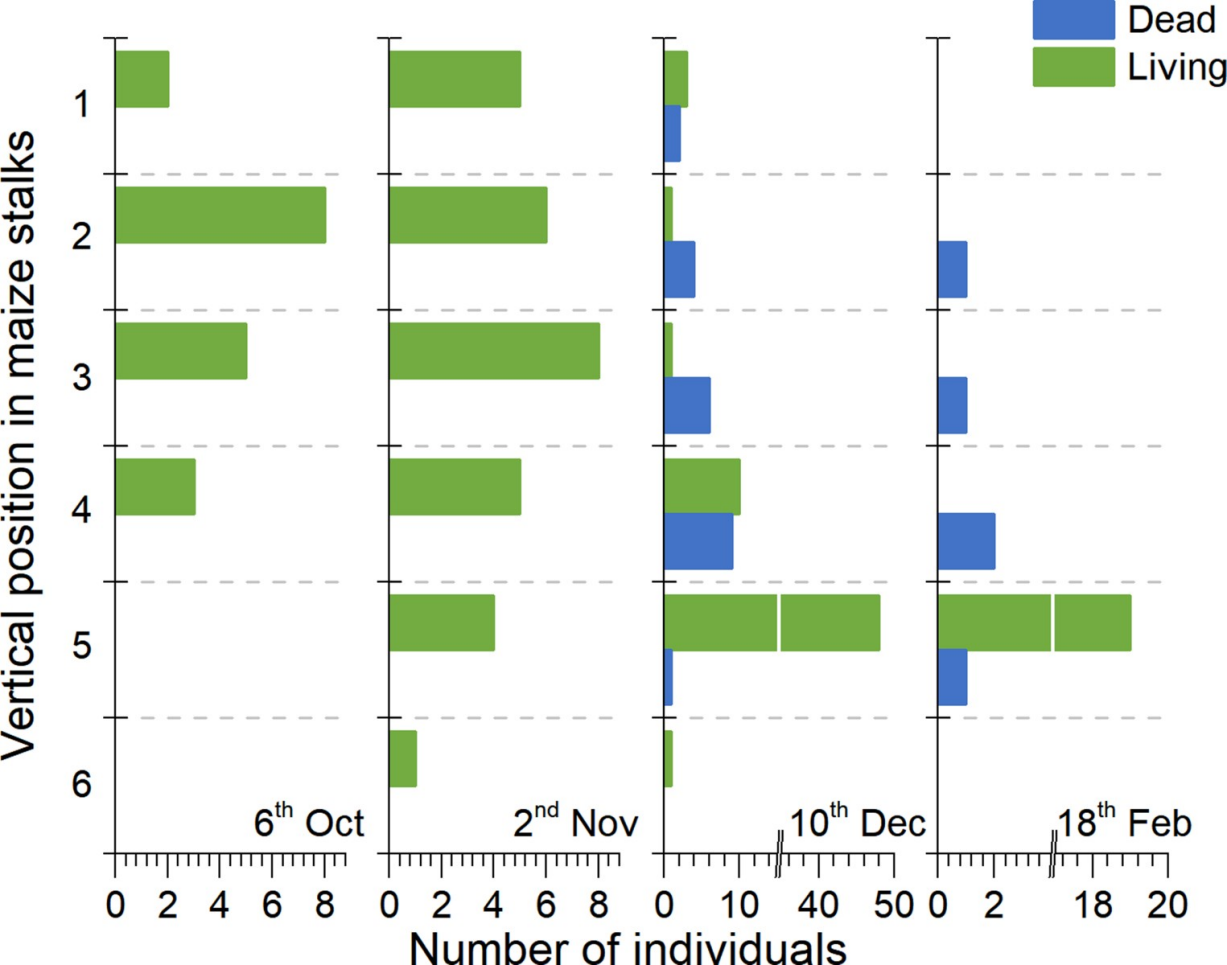
Underground-forward behavior of *S*. *inferens* in remaining summer corn stalks in winter.

**Fig 3:**
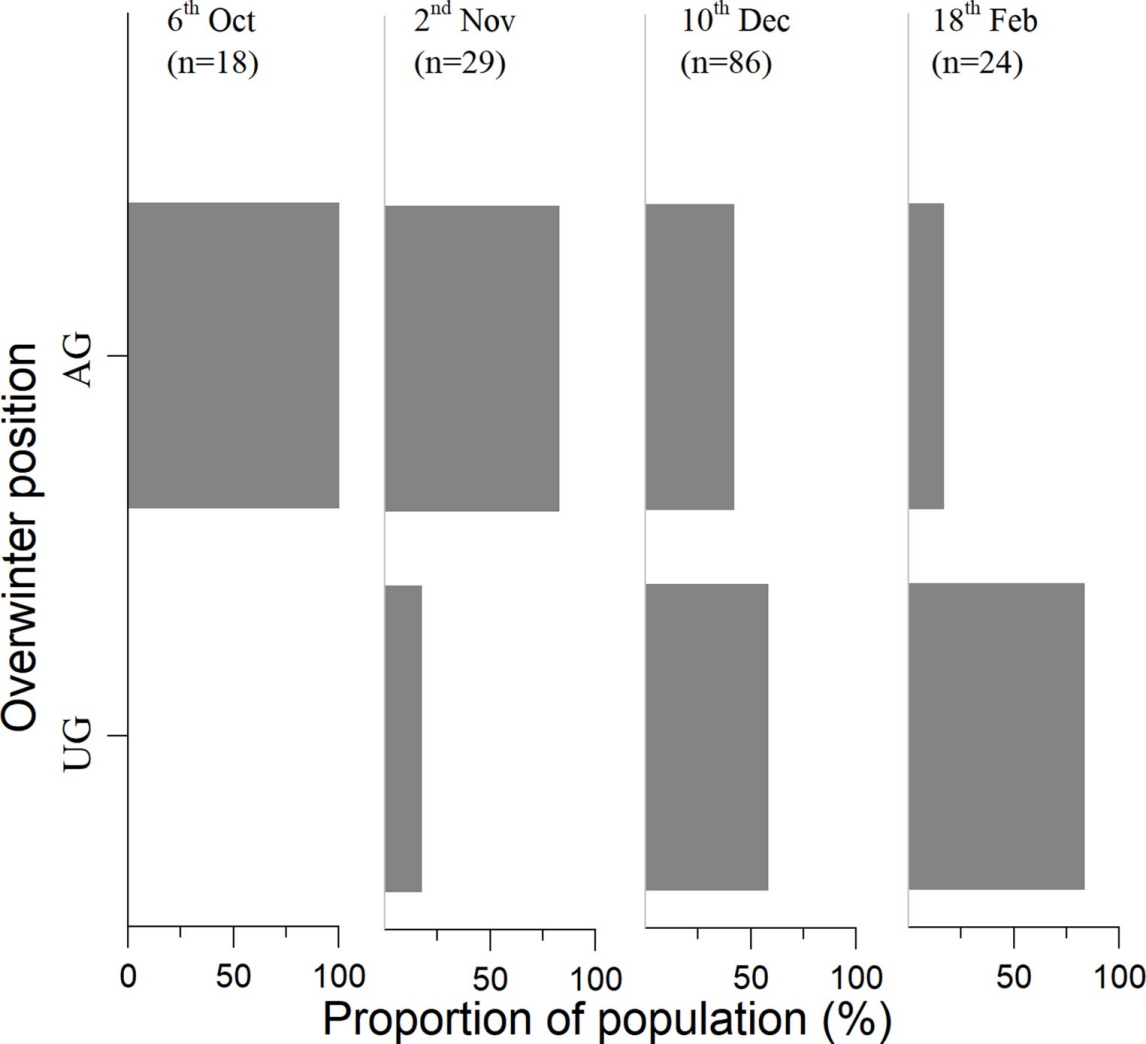
Proportion of *S*. *inferens* population (dead and alive) in overwintering positions in XX. UG: Underground; AG: above ground.

In early winter (October 6^th^ and November 2^nd^), 100% of the *S*. *inferens* population was alive and the highest numbers of insects were at positions 2 and 3. In later winter (December 10^th^ and February 18^th^), the incidences of population mortality were 25.5% and 20.83% respectively, and the highest numbers of insects were both at position 5 (Fig 2). In the early winter, most of the *S*. *inferens* population was distributed above ground. The location of the whole population shifted to the ground when the winter became colder, and most (83.3%) of the *S*. *inferens* population (dead and alive) was distributed underground by the 18^th^ of February (Fig 3). On the 10^th^ of December, the ratio of dead individuals above ground and underground was 21:1 and on the 18^th^ of February, it was 4:1. All of the living individuals were only found at underground position 5 and all the remaining population above ground in winter were dead by February 18^th^ (Fig 2).

### 3.3 Cold hardiness of *S*. *inferens*

**Fig 4:**
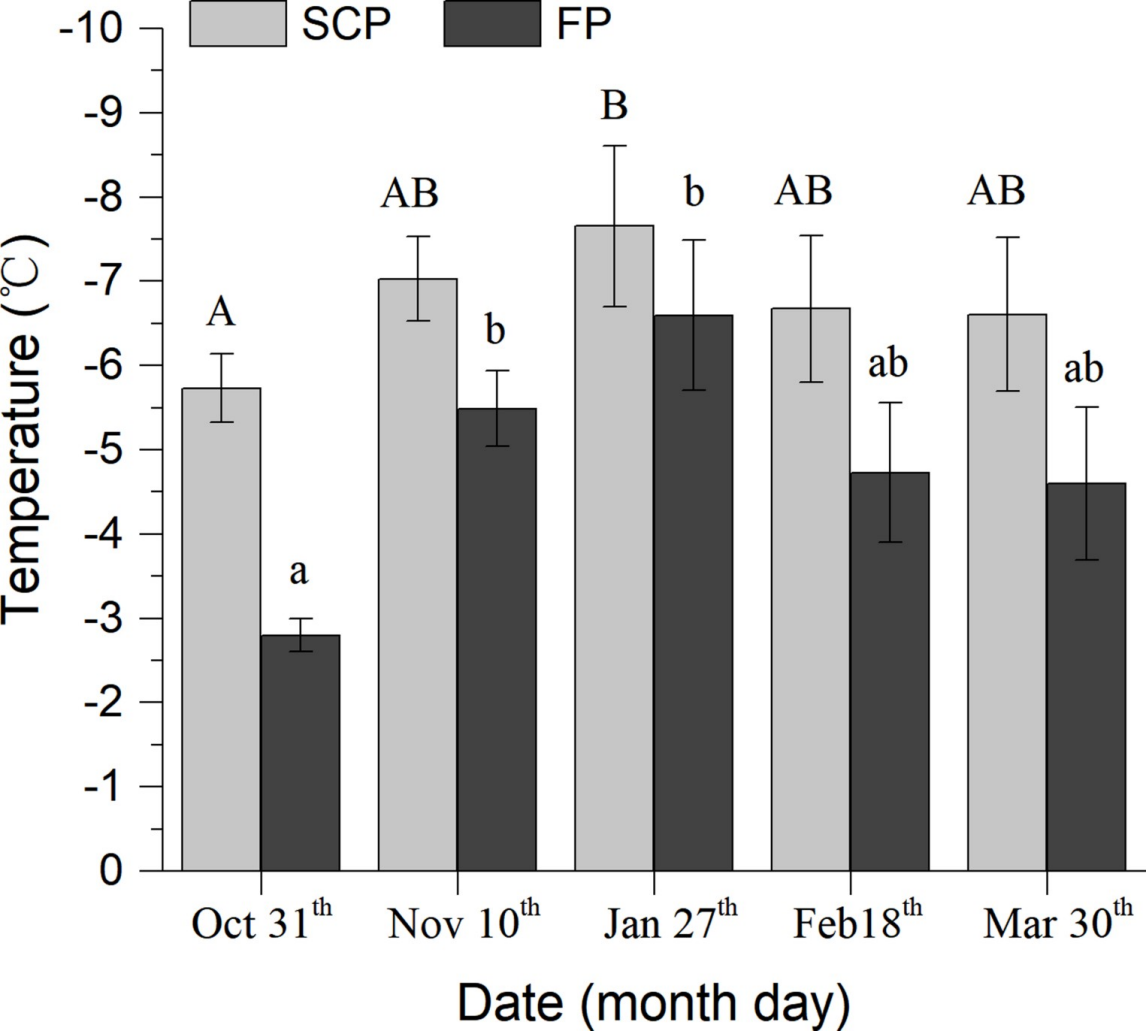
SCP and FP of *S*. *inferens* larvae during the overwinter period. Same pattern bar with different letters on top shows a significant difference at *p*< 0.05.

There were significant differences in SCP and FP of *S*. *inferens* larvae over time (SCP: *F*_4,91_ = 4.993, *p* = 0.003; FP: *F*_4,91_ = 10.26, *p*<0.001). The mean SCP (±SE) of *S*. *inferens* larvae ranged from the highest (-5.731±0.405°C) in October to the lowest (-7.653±0.952°C) in January (Fig 4) and the mean and minimum value of the SCP first decreased and then rose with the cold temperature event. The lowest value -20.02°C appeared on the 27^th^ of Jan (Table 1). Similarly, the mean of the FP first decreased and then rose. The means of the FPs in middle winter (December: -5.49±0.447; January: -6.596±0.889) were significantly lower than early and end of winter (November, October and March). In contrast, there was no significant difference in the fresh weight of larvae during the cold temperature period (*F*_4,91_ = 1.997, *p* = 0.102), but there was a significant gradual decrease in dry weight (*F*_4,91_ = 3.934, *p* = 0.006) and water content of the larvae (*F*_4,87_ = 3.19, *p* = 0.017). Larvae dry weight showed a decreased tendency throughout the winter and the water content decreased at the beginning of winter and slightly increased at the end of winter (Table 1).

**Table 1:** Minimum SCP, fresh mass, dry mass, water content and the incidence of mortality of *S*. *inferens* larvae after SCP test.

Date	*n*	Minimum SCP (°C)	Fresh mass (g)	Dry mass (g)	Water content (g)	Incidence of mortality after cold hardiness test (%)	Daily mean air temperature (°C)
Oct 31^st^	24	-10.38	0.19±0.015 a	0.069±0.007 ab	0.121±0.008 a	91.667	13.67
Dec 10^th^	20	-11.95	0.179±0.019 a	0.087±0.012 a	0.092±0.01 ab	30	2.18
Jan 27^th^	19	-20.02	0.146±0.016 a	0.051±0.007 bc	0.094±0.01 ab	15.789	-3.24
Feb 18^th^	12	-19.74	0.157±0.02 a	0.069±0.011 ab	0.088±0.01 b	37.5	3.03
Mar 31^st^	17	-17.9	0.14±0.011 a	0.037±0.005 c	0.103±0.007 ab	76.471	20.19

(SCP: Supercooling points. Means flanked with different letters at the same column are significantly different at *P*<0.05).

After a 30 minute SCP test, 91.667% of the tested *S*. *inferens* larvae were dead when returned to 24°C immediately after freezing on October 31^st^. About sixteen percent died in the January 27^th^ SCP test, and 76.471% died in the March 31^st^ test. The number of dead larvae after the test was significantly negatively correlated with the daily air mean temperature on the sampling date (*r* = -0.915, *p* = 0.029) (Table 1). The species was therefore determined to be a moderately freeze-tolerant insect.

### 3.4 Current and potential distribution of *S*. *inferens*

**Fig 5:**
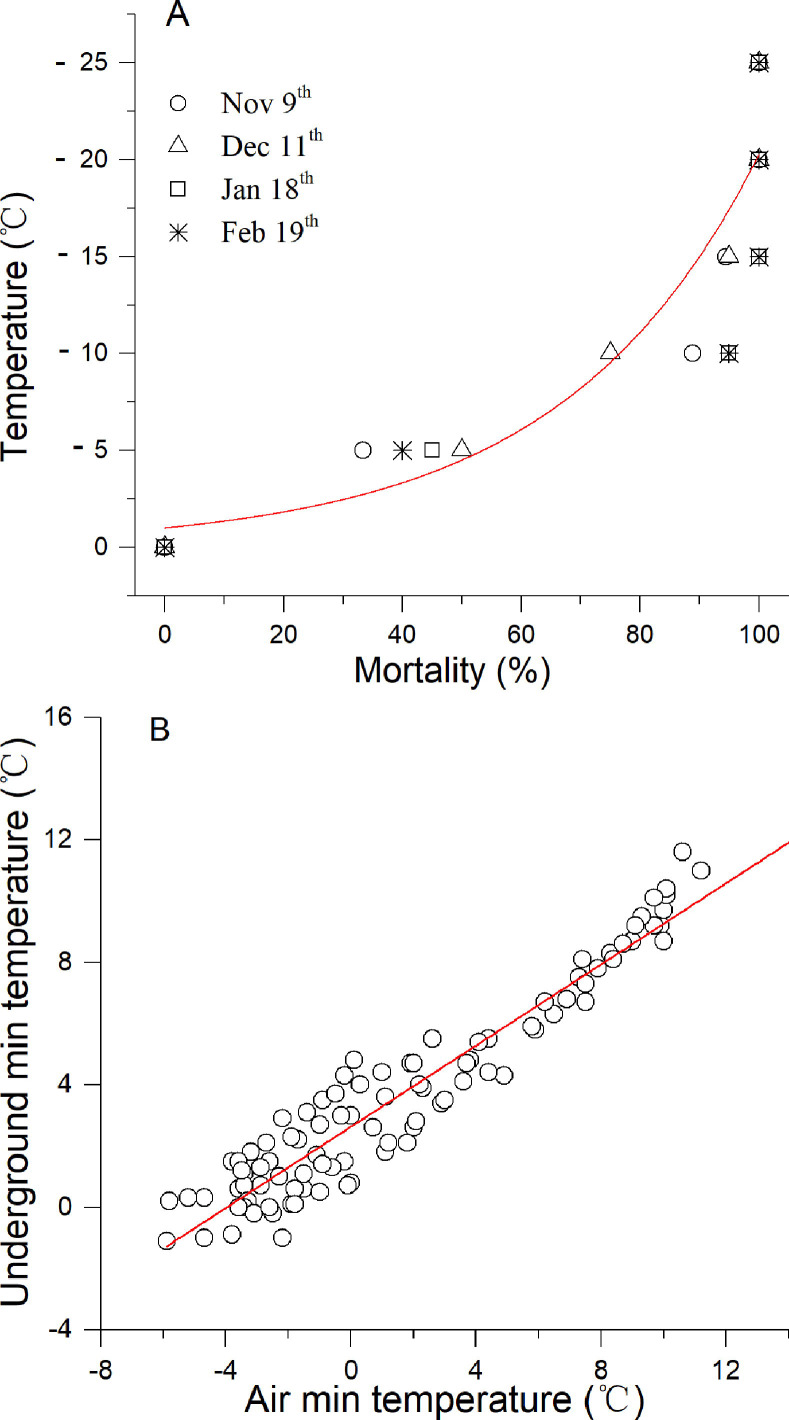
(A) The incidence of mortality of *S*. *inferens* larvae after lethal low temperature experiments and (B) the relationship between daily minimum air and underground temperature during the overwinter period.

**Fig 6:**
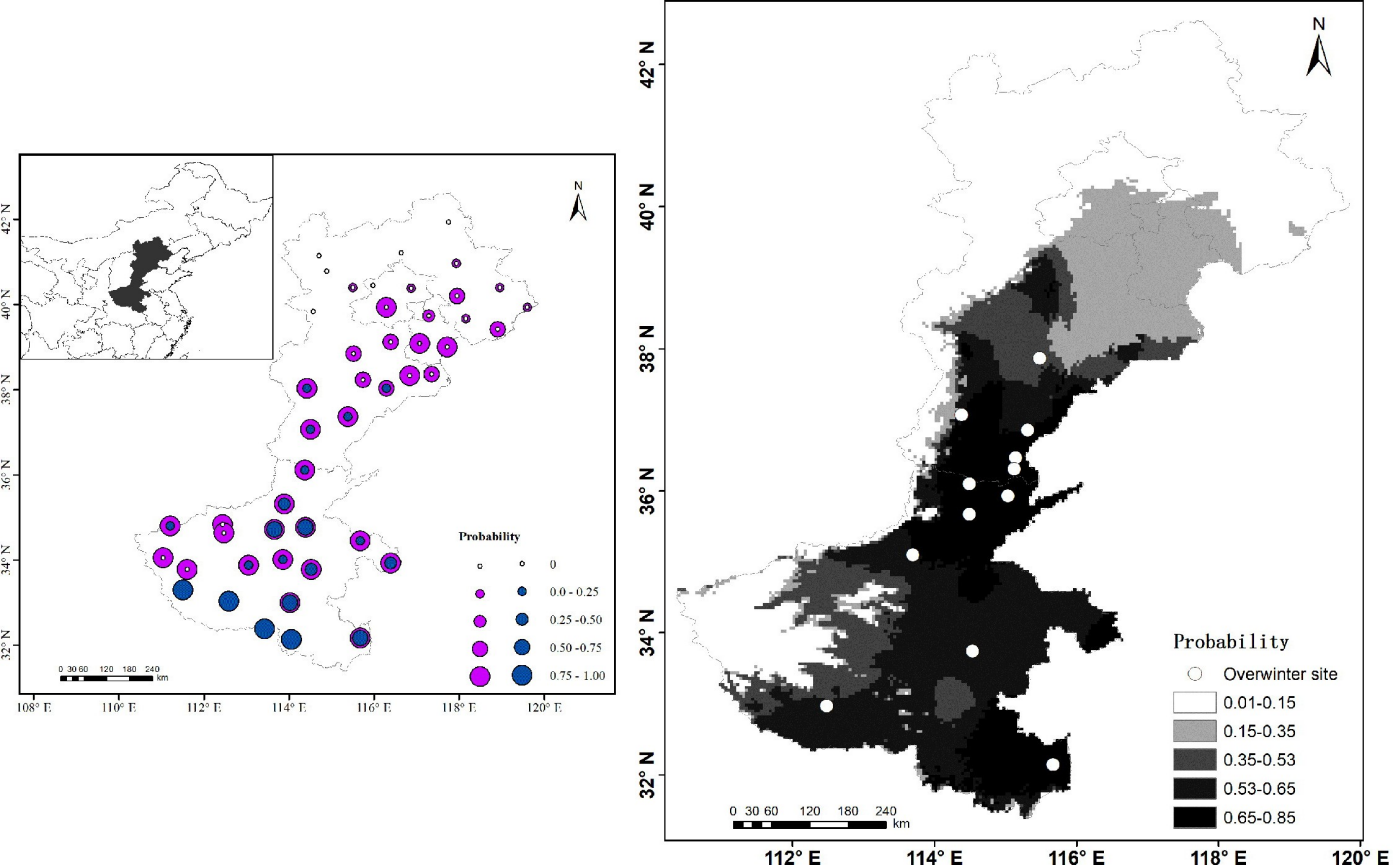
Current and potential distribution of *S*. *inferens* in northern China. (A) Potential distribution based on the low temperature threshold, the size of the circle in A means the survival probability, and the blue and purple circles are sourced from ALLT_50_ and ULLT_50_, respectively. (B) Current overwinter sites and predicted suitable habitat area modeling by MaxEnt. The darker color in B represents the better predicted habitat suitability. The white points are the new northern overwinter sites. The map was produced using ArcGIS 10.2 under a CC BY license, with permission from ESRI (www.esri.com) and shapefile reprinted from GADM database under a CC BY license, with permission from Global Administrative Areas (www.gadm.org).

There were no significant differences between different sampling periods of *S*. *inferens* in winter (*F*_*3*,*24*_ = 0.002, *p* = 0.998), but there were significant differences between the length of time the insects were exposed to subzero temperatures (*F*_*1*,*24*_ = 19.650, *p* = 0.002). Therefore, we combined the dataset and only considered the effects of exposure temperatures. The ALLT_50_ was -4.5 based on a nonlinear regression: *y* = (1.03054)^*x*^ (*y*: Incidence of mortality, *x*: Temperature (°C), *R*^2^ = 0.878, *p*<0.001, *n* = 24) (Fig 5A). The ULLT_50_ was -9.713°C after being translated by a linear model between minimum daily air and underground temperature: *y =* 0.6628*x+2.6267 (*y*: Underground, *x*: Air, *R*^2^ = 0.9161, *p*<0.0001, *n* = 100) (Fig 5B). The regional survival ratio was calculated with both ALLT_50_ and ULLT_50_ based on low temperature threshold. ≥50% probability survival regions were below N 35° due to the ALLT_50_ and below N 40° due to the ULLT_50_ (Fig 6A).

The MaxEnt model predicted potential distribution of *S*. *inferens* with a high accuracy test AUC value of 0.97 and training AUC value of 0.981 (S2 Fig), and three temperature-variables: BIO6 (minimum temperature of coldest month), BIO10 (mean temperature of warmest quarter), and BIO2 (mean of monthly difference of maximum and minimum temperature), which showed a top three permutation importance on the model (S3 Table). Model predictions closely matched the new overwinter site and also showed potentially suitable districts in north regions, and predicted suitable habitat areas of *S*. *inferens* below N 40°. The most suitable areas were below N 38° (Fig 6B). This means that the underground overwinter temperature boundary defined by the ULLT_50_ is reliable, and overwinter strategies preventing winterkilling led to five latitude wider overwintering regions.

## 4 Discussion

There are many studies which show the latitudinal pattern of insects facing cold temperatures in winter. Higher latitude populations possess a stronger cold tolerance, a shorter chill-coma recovery time and a higher survival rate [29–34]. This research shows the overwintering location of the *S*. *inferens* population was closer to the ground with increasing latitude. In the new northern breeding area in winter, with the temperature decreasing, *S*. *inferens* population gradually climbed down and drilled into the base of the maize stalk under the soil surface before the coldest temperature arrived. Larvae remaining above ground in winter were all winterkilled in later winter. Compared to the year-round site at N 19° in the south [35], it is an obvious behavioral strategy of *S*. *inferens* to survive, to find the warmer microclimate of the overwintering site in the north in winter. The different latitudinal position and underground-forward behavior observed may be entirely lower temperature driven. A further indoor experimental test is needed to confirm that populations from the south exhibit the same behavior if exposed to colder temperatures, so that a population behavior could be defined. Insects usually select warmer micro-habitats to survive. *Coccinella septempunctata* L., *Ceratomegilla undecimnotata* (Schneider), *Hippodamia variegata* (Goeze) *Harmonia axyridis* (Pallas) and *Coccinella septempunctata* select human houses, isolated grass tussocks or under covered stones as overwintering sites [36, 6, 37]. Similarly, *Sesamia nonagrioide*, the same family of the insects in the study but another species, also show underground-forward behavior in winter in Landes (South-west of France, 45°N, 0.7°W) [38].

Physiological adaptation of insects for low temperatures is by partial dehydration, increasing body fluid osmolality, and the accumulation of a complex mixture of winter specific metabolites, which strengthen their cold hardiness [39, 37]. In this paper, the mean SCP of larvae which were collected from the field was -7.653±0.952°C (lowest), and the incidence of mortality of the population was 15.789% (lowest) after a 30 minute cold hardiness test in January (middle winter). Meanwhile, the water contents gradually decreased and were lowest on Feb 18^th^ which shows the diapaused insects transfer fluidity into antifreeze proteins and glycoproteins [40]. When insects begin to recover in spring the fluidity increases. In this research, insects survived when body temperature was under freezing point and showed an extreme low temperature tolerance capability meaning that the population of *S*. *inferens* larvae in the northern breeding area was a moderately freeze-tolerant one. These results correspond with Guo et al. [17] and Sun [18] where the pest hosts on rice. Cold hardiness is also related to the host insects have fed on [41, 42] and the differences in the cold tolerance mechanisms between maize and rice need to be further investigated in the new breeding region.

Minimum temperatures in the coldest month are linearly related to insect mortality [29]. ALLT_50_ or the lethal temperature (LTe50) of insect cold exposure are the best predictors of cold distribution limits and are usually used to assess the potential distribution according to the annual minimum temperature [43, 23]. In this research, the ALLT_50_ and ULLT_50_ were -9.7°Cand -4.5°C, respectively. The method particularly included the effect of different exposure durations in different winter periods. In natural conditions, there were two days when the daily mean temperatures in the soil were subzero in 2015–2016 in the middle-south district (Xinxiang), but in north, there were more subzero days. Therefore, the larvae in the field will experience different durations of subzero temperatures in the soil in different latitudinal places. The ALLT_50_ we identified was the subzero temperature effect after experiencing different exposure durations; the value was -9.7°C. This is more realistic than the lower ALLT_50_ (-6.12°C) of larvae which was sourced through consistent exposure duration (2 h) collected on rice in Oct 2012 in Yangzhou by Sun [18], particularly when assessing the distribution of insects on a large scale in winter. Other research also shows that short-term lower temperature interruption procedures causes higher death and results in a higher ALLT_50_ [44]. This study used the lower temperature criteria which discriminated by up or below the ALLT_50_/ULLT_50_ in annual daily minimum temperatures, and show a northern distribution considering the ALLT_50_ in locations under 35°N. However, depending on the ULLT_50_, the results showed a northerly distribution below 40°N when insects overwinter underground. This was also in agreement with the results of the MaxEnt modeling, which show below 40°N predicted suitable habitat areas. Distribution of insects is highly impacted by climatic factors (temperature, moisture, humidity and their variations), especially the effects of temperature [45]. MaxEnt integrates insect occurrence records with climatic and other environmental variables and the MaxEnt model of this study shows a similar result to de la Vega [46], that the minimum temperature of the coldest month was the important abiotic factor restricting the geographic distribution of *Triatoma infestans* and *Rhodnius prolixus*. The MaxEnt model also included many other important parameters such as the precipitation, which showed a high contribution in the model. It is usually used to predict potential distributions of insect pests [27]. Although artificial because the linear model based on the point data might not represent the actual regional patterns, the linear relationship between air and soil temperature has indeed been studied in different areas [47, 48]. Hong [24] has taken advantage of this linear relationship from 96 weather stations in Shanxi, China, and classified the overwintering sites of the southern root-knot nematode. These results also show that the underground low temperature threshold defined by the ULLT_50_ could be extrapolated to other underground overwinter species.

The population behavior of animals which escape cold weather usually by large- scale migration into a warmer habitat, will lead to relocated populations regionally, but not all individuals can successfully find suitable places. *S*. *inferens* have a weak flight tendency and capability in the field [49]. This paper shows another behavioral strategy of *S*. *inferens* larvae by locally searching for a warmer place by drilling into the ground in early winter. The microclimate of the overwintering site could result in the *S*. *inferens* colonizing more northerly latitudes and the insect’s physiological and biochemical changes may be a factor favoring this northern expansion leading to a higher winter survival chance. Past studies have shown that *S*. *inferens* are distributed below N 34° degrees north latitude. This study also documents a new location for *S*. *inferens*: a northern distribution record of N 40°. The overwintering boundary of many insects has also been reportedly spreading poleward due to global warming [50]. It cannot be overlooked that the warmer changes in northern China in these two centuries have driven the change of animal redistribution [51], but the strategy of behavioral or physiological changes in this study to overcome winter will certainly help to colonize a five degree more northerly latitude. For maize producers, ploughing the maize stubble in autumn after harvesting will lead to an increased mortality of *S*. *inferens* larvae due to cold exposure. This study allows us to propose a simple non-pollutant pest control method in northern China.

## Supporting information

S1 Fig The Receiver Operating Characteristic (ROC) curve of the MaxEnt model. (DOCX)

S2 Fig AUC of different environmental variables based on results of jackknife tests in the MaxEnt model. (DOCX)

S1 Table The live number of *S*. *inferens* larvae at northern overwinter sites. (DOCX)

S2 Table The worldwide distribution of *S*. *inferens*. (DOCX)

S3 Table The permutation importance and relative contributions of the environmental variables in the MaxEnt model. (DOCX)
