# Supplementary figures and images for: Low-temperature derived temporal change in the vertical distribution of Sesamia inferens larvae in winter, with links to its latitudinal distribution
Source: PLoS One. 2020 Jul 28;15(7):e0236174. doi: 10.1371/journal.pone.0236174 (PMC7386632; doi:10.1371/journal.pone.0236174)

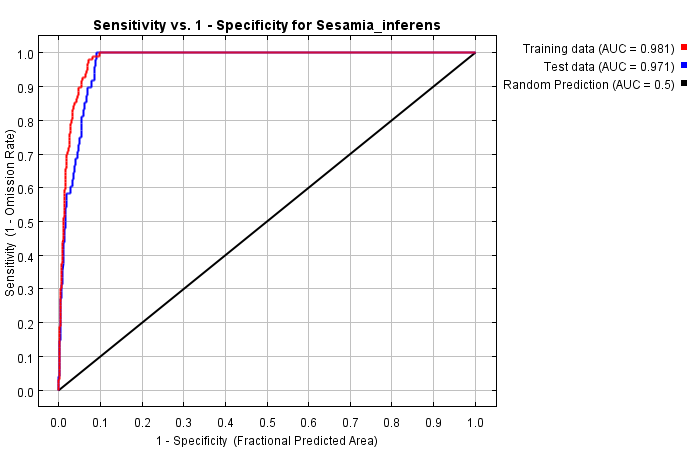


**Figure S1.**  **The receiver operating characteristic (ROC) curve of the MaxEnt model.**

Supplement: S1 Fig — (DOCX) [file pone.0236174.s001.docx]
