# Supplementary material for: Low-temperature derived temporal change in the vertical distribution of Sesamia inferens larvae in winter, with links to its latitudinal distribution
Source: PLoS One. 2020 Jul 28;15(7):e0236174. doi: 10.1371/journal.pone.0236174 (PMC7386632; doi:10.1371/journal.pone.0236174)
Supplement: S2 Fig — (DOCX) [file pone.0236174.s002.docx]

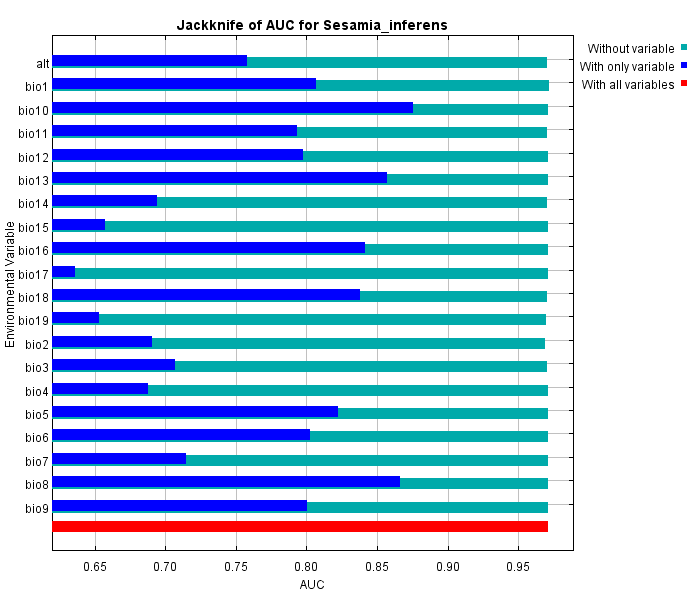


**Figure S2.** **AUC of different environmental variables based on results of jackknife tests in the MaxEnt model.**
