# Supplementary material for: Low-temperature derived temporal change in the vertical distribution of Sesamia inferens larvae in winter, with links to its latitudinal distribution
Source: PLoS One. 2020 Jul 28;15(7):e0236174. doi: 10.1371/journal.pone.0236174 (PMC7386632; doi:10.1371/journal.pone.0236174)
Supplement: S1 Table — (DOCX) [file pone.0236174.s003.docx]

**Table S1.** **The alive number of *S. inferens* larvae in northern overwinter sites.**

| City names | Latitude | Longitude | Alive rate (%) | Death rate(%) | Alive number | Total Number |
| --- | --- | --- | --- | --- | --- | --- |
| XUX | 114.4995 | 35.694 | 66.66667 | 33.33333 | 6 | 15 |
| QF | 115.0132 | 35.9132 | 64.28571 | 35.71429 | 9 | 23 |
| HDS | 115.1145 | 36.4699 | 38.46154 | 61.53846 | 5 | 18 |
| HDQ | 115.2345 | 36.8103 | 20 | 80 | 2 | 12 |
| NG | 115.4359 | 37.3363 | 20 | 80 | 1 | 6 |
| XT | 114.5439 | 37.1053 | 28.57143 | 71.42857 | 4 | 18 |
| AY | 114.446 | 36.1076 | 57.14286 | 42.85714 | 4 | 11 |
| XX | 113.7042 | 35.01662 | 73.68421 | 26.31579 | 19 | 25 |

(XUX: Xunxian, QF: Qingfeng, HDS: Handanshatayi, HDQ: Handanqiuxian, NG: Nangong, XT: Xingtai, AY: Anyang, XX:Xingxiang )
