# Supplementary material for: Low-temperature derived temporal change in the vertical distribution of Sesamia inferens larvae in winter, with links to its latitudinal distribution
Source: PLoS One. 2020 Jul 28;15(7):e0236174. doi: 10.1371/journal.pone.0236174 (PMC7386632; doi:10.1371/journal.pone.0236174)
Supplement: S2 Table — (DOCX) [file pone.0236174.s004.docx]

**Table S2.** **The worldwide distribution of *S. inferens***

| Longitude | Latitude | Country | City |
| --- | --- | --- | --- |
| 120.722 | 15.482 | Philippines | Central Luzon |
| 120.922 | 14.396 | Philippines | Manila |
| 119.93 | 30.075 | China Mainland | Zhejing |
| 118.875 | 32.03 | China Mainland | Nanjing |
| 109.54 | 19.493 | China Mainland | Shanzhou |
| 107.162 | 23.061 | China Mainland | Tiandeng |
| 106.579 | 24.359 | China Mainland | Lingyun |
| 112.426 | 23.131 | China Mainland | Zhaoqin |
| 113.089 | 22.426 | China Mainland | Xinhui |
| 107.804 | 26.181 | China Mainland | Danzhai |
| 108.515 | 28.588 | China Mainland | Ruanhe |
| 119.3 | 26.1 | China Mainland | Fuzhou |
| 117.976 | 24.554 | China Mainland | Xiamen |
| 103.259 | 23.678 | China Mainland | Honghe |
| 103.864 | 25.495 | China Mainland | Qujing |
| 103.311 | 23.607 | China Mainland | Kaiyuan |
| 104.623 | 28.731 | China Mainland | Yibing |
| 104.314 | 30.969 | China Mainland | Guanghan |
| 102.861 | 30.006 | China Mainland | Yaan |
| 105.856 | 32.409 | China Mainland | Guangyuan |
| 112.829 | 32.132 | China Mainland | Zaoyang |
| 112.215 | 31.02 | China Mainland | Jingmen |
| 109.437 | 30.334 | China Mainland | Enshi |
| 112.022 | 32.037 | China Mainland | Xiangyang |
| 108.44 | 30.904 | China Mainland | Wanzhou |
| 119.325 | 32.313 | China Mainland | Yangzhou |
| 120.905 | 31.353 | China Mainland | Kunshan |
| 120.334 | 32.559 | China Mainland | Haian |
| 120.096 | 33.262 | China Mainland | Yanchen |
| 118.904 | 33.504 | China Mainland | Huaian |
| 121.057 | 31.995 | China Mainland | Tongzhou |
| 121.36 | 30.935 | China Mainland | Qingpu |
| 116.117 | 28.71 | China Mainland | Nanchang |
| 114.383 | 25.377 | China Mainland | Ganzhou |
| 112.996 | 25.786 | China Mainland | Chenzhou |
| 113.013 | 29.053 | China Mainland | Dongting |
| 120.975 | 30.655 | China Mainland | Jiaxing |
| 120.717 | 28.868 | China Mainland | Xianju |
| 119.625 | 27.97 | China Mainland | Jingning |
| 116.909 | 31.46 | China Mainland | Liuan |
| 118.257 | 29.736 | China Mainland | Huangshan |
| 117.9 | 31.561 | China Mainland | Chaohu |
| 118.488 | 31.345 | China Mainland | Wuhu |
| 117.656 | 32.514 | China Mainland | Chuzhou |
| 115.745 | 32.854 | China Mainland | Fuyang |
| 117.12 | 34.165 | China Mainland | Xuzhou |
| 119.395 | 34.606 | China Mainland | Lianyungang |
| 113.695 | 35.093 | China Mainland | Xinxiang |
| 114.49 | 35.666 | China Mainland | Hebi |
| 115.03 | 35.928 | China Mainland | Puyang |
| 115.121 | 36.302 | China Mainland | Daming |
| 115.139 | 36.464 | China Mainland | Guantao |
| 115.31 | 36.85 | China Mainland | Qiuxian |
| 114.489 | 36.095 | China Mainland | Anyang |
| 114.379 | 37.065 | China Mainland | Xingtai |
| 114.534 | 33.739 | China Mainland | Zhoukou |
| 115.876 | 36.957 | China Mainland | Xiajing |
| 115.848 | 36.426 | China Mainland | Liaochen |
| 117.831 | 37.362 | China Mainland | Bingzhou |
| 116.825 | 36.191 | China Mainland | Taian |
| 115.481 | 37.865 | China Mainland | Hengshui |
| 126.909 | 35.789 | Korea | North Jeolla |
| 128.897 | 35.206 | Korea | South Gyeongsang |
| 115.668 | 32.144 | China Mainland | Gushi |
| 112.477 | 32.969 | China Mainland | Nanyang |
| 120.208 | 22.892 | Taiwan | Tainan |
| 76.66 | 12.382 | India | Mysore |
| 75.726 | 15.317 | India | Karnataka |
| 84.33 | 27.586 | Nepal | Chitwan |
| 79.061 | 28.801 | India | Rampur |
| 110.314 | 1.54 | Malaysia | Sarawak |
| 127.989 | 26.514 | Japan | Ryukyus |
| 129.305 | 28.273 | Japan | Amami-Group |
| 114.829 | 4.872 | Brunei | Suasa |
| 116.063 | 5.903 | Malaysia | Sarawak |
| 127.772 | 26.354 | Japan | Okinawa |
| 73.161 | 33.731 | Pakistan | Islamabad |
| 67.652 | 24.58 | Pakistan | Kachhla |
| 121.313 | 14.143 | Philippines | Laguna |
| 130.475 | 31.571 | Japan | Kagoshima |
| 75.393 | 11.884 | India | Kannur |
| 76.353 | 10.307 | India | Kerala state |
| 127.997 | 26.394 | Japan | Ikei Island |
| 110.72 | -7.619 | Indonesia | Java |
| 103.895 | -3.32 | Indonesia | South Sumatera |
| 115.223 | -3.106 | Indonesia | South Kalimantan |
| 115.196 | -8.419 | Indonesia | Bali |
| 120 | -3.662 | Indonesia | South Sulawesi |
| 68.546 | 25.408 | Pakistan | Tandojam |
| 75.544 | 30.554 | India | Ludhiana |
| 88.656 | 25.625 | Bangladesh. | Dinajpur |
| 88.648 | 24.374 | Bangladesh | Rajshahi |
| 77.646 | 12.939 | Indian | Bangalore |
| 72.868 | 19.162 | Indian | Sandoz India |
| 73.091 | 31.251 | Pakistan | Faisalabad |
| 76.917 | 29.798 | Indian | Taraori |
| 86.021 | 24.486 | Indian | Basmati |
| 76.846 | 30.737 | India | Uttar |
| 75.712 | 15.307 | India | Karnataka |
| 74.185 | 27.028 | Indian | Rajasthan |
| 75.346 | 31.148 | Indian | Punjab |
| 110.345 | 1.542 | Malaysian | Sarawak |
| 78.37 | 17.286 | India | Hyderabad |
| 77.112 | 28.591 | Indian | New Delhi |
| 120.341 | 30.341 | China Mainland | Hangzhou |
| 100.696 | 13.887 | Thailand | Bangkhen |
| 80.018 | 13.028 | Indian | Madras |
| 85.103 | 20.944 | India | Orissa State |
| 80.959 | 26.845 | India | Lucknow |
| 80.759 | 7.881 | Sri Lanka | Matale |
| 160.193 | -9.488 | Solomon Islands | Solomon Islands |
| 120.817 | 24.009 | Taiwan(China) | Formosa |
| 136.887 | -4.569 | New Guinea Islands | New Guinea Islands |
| 108.039 | 14.038 | Vietnam | South Vietnam |
| 105.32 | 21.287 | Vietnam | Ha Noi |
| 119.812 | -3.87 | Indonesia | South Sulawesi |
| 99.948 | 14.036 | Thailand | Kampaeng Saen |
| 100.05 | 13.76 | Thailand | Pathom |
| 99.127 | 14.116 | Thailand | Sai Yok |
| 112.247 | -7.527 | Indonesia | Jawa Timur |
| 80.988 | 26.827 | India | Uttar Pradesh |
| 86.778 | 25.927 | India | Madhipura |
| 88.456 | 27.064 | India | Kalimpong |
| 80.986 | 26.824 | India | West Bengal |
| 85.101 | 20.947 | India | Orissa |
| 85.324 | 25.089 | India | Orissa |
| 77.746 | 22.729 | India | Hoshangabad |
| 77.58 | 13.018 | India | Hebbal |
| 78.681 | 11.13 | India | Tamil Nadu |
| 87.853 | 22.953 | India | Bengal |
| 121.479 | 14.14 | Philippines | Laguna |
| 142.34 | -7.922 | Papua new guinea | Western |
| 141.695 | -3.726 | Papua new guinea | Sundaun |
| 146.875 | -2.089 | Papua new guinea | Manus |
| 152.943 | -4.288 | Papua new guinea | New Ireland |
| 151.75 | -4.512 | Papua new guinea | New britain |
| 92.975 | 13.228 | India | Andaman islands |
| 87.86 | 22.927 | India | West Bengal |
| 114.988 | 0.885 | Malaysia | Borneo |
| 95.387 | 23.592 | Burma | Sagaing |
| 104.813 | 12.652 | Cambodia | Kampong Thum |
| 80.752 | 7.873 | Ceylon | Matale |
| 88.437 | 27.265 | India | Sikkim |
| 103.716 | 1.437 | Singapore | North |
| 110.517 | -7.054 | Indonesia | Irian |
| 119.895 | -1.096 | Indonesia | Pulu |
