# Supplementary material for: Low-temperature derived temporal change in the vertical distribution of Sesamia inferens larvae in winter, with links to its latitudinal distribution
Source: PLoS One. 2020 Jul 28;15(7):e0236174. doi: 10.1371/journal.pone.0236174 (PMC7386632; doi:10.1371/journal.pone.0236174)
Supplement: S3 Table — (DOCX) [file pone.0236174.s005.docx]

**Table S3.** **The permutation importance and relative contributions of the environmental variables in the MaxEnt model**

| **Variable** | **represent** | **Permutation importance** | **Percent contribution** |
| --- | --- | --- | --- |
| BIO2 | Mean of Monthly Difference of Maximum - Minimum Temperature | 27.8 | 7.9 |
| BIO10 | Mean Temperature of Warmest Quarter | 18.1 | 10.1 |
| BIO6 | Min Temperature of Coldest Month | 10.3 | 12.1 |
| BIO13 | Precipitation of Wettest Month | 8.2 | 31.5 |
| BIO8 | Mean Temperature of Wettest Quarter | 9.6 | 16.1 |
| BIO3 | Isothermality (BIO2/BIO7) (*100) | 6.2 | 13.7 |
| BIO18 | Precipitation of Warmest Quarter | 4.5 | 6.1 |
| BIO7 | Temperature Annual Range (BIO5-BIO6) | 0.3 | 1 |
